# Predicting Transcriptional Activity of Multiple Site p53 Mutants Based on Hybrid Properties

**DOI:** 10.1371/journal.pone.0022940

**Published:** 2011-08-08

**Authors:** Tao Huang, Shen Niu, Zhongping Xu, Yun Huang, Xiangyin Kong, Yu-Dong Cai, Kuo-Chen Chou

**Affiliations:** 1 Institute of Systems Biology, Shanghai University, Shanghai, People's Republic of China; 2 Key Laboratory of Systems Biology, Shanghai Institutes for Biological Sciences, Chinese Academy of Sciences, Shanghai, People's Republic of China; 3 Shanghai Center for Bioinformation Technology, Shanghai, People's Republic of China; 4 Centre for Computational Systems Biology, Fudan University, Shanghai, People's Republic of China; 5 Key Laboratory of Stem Cell Biology, Institute of Health Sciences, Shanghai Institutes for Biological Sciences, Chinese Academy of Sciences and Shanghai Jiao Tong University School of Medicine, Shanghai, People's Republic of China; 6 State Key Laboratory of Medical Genomics, Ruijin Hospital, Shanghai Jiaotong University, Shanghai, People's Republic of China; 7 Gordon Life Science Institute, San Diego, California, United States of America; Reiner Albert Veitia, Institute Jacques Monod, France

## Abstract

As an important tumor suppressor protein, reactivate mutated p53 was found in many kinds of human cancers and that restoring active p53 would lead to tumor regression. In this work, we developed a new computational method to predict the transcriptional activity for one-, two-, three- and four-site p53 mutants, respectively. With the approach from the general form of pseudo amino acid composition, we used eight types of features to represent the mutation and then selected the optimal prediction features based on the maximum relevance, minimum redundancy, and incremental feature selection methods. The Mathew's correlation coefficients (MCC) obtained by using nearest neighbor algorithm and jackknife cross validation for one-, two-, three- and four-site p53 mutants were 0.678, 0.314, 0.705, and 0.907, respectively. It was revealed by the further optimal feature set analysis that the 2D (two-dimensional) structure features composed the largest part of the optimal feature set and maybe played the most important roles in all four types of p53 mutant active status prediction. It was also demonstrated by the optimal feature sets, especially those at the top level, that the 3D structure features, conservation, physicochemical and biochemical properties of amino acid near the mutation site, also played quite important roles for p53 mutant active status prediction. Our study has provided a new and promising approach for finding functionally important sites and the relevant features for in-depth study of p53 protein and its action mechanism.

## Introduction

As a critical tumor suppressor gene, p53 plays an important role in maintaining genomic stability and preventing cancer [1], [2], [3]. It has the highest mutation frequency in human tumors: over 50% of kinds of tumors have p53 mutations, and over 80% of kinds of tumors involve dysfunctional p53 signaling pathway [4]. It was reported that restoring p53 activity could lead to tumour regression and that p53 mutants could be reactivate in vivo through intragenic second-site suppressor mutations. In view of this, it is worthwhile for us to conduct an in-depth study on the occurrence of p53 mutation because the findings thus obtained may provide useful insights for developing new drugs that possess similar functions of “cancer rescue” via mutation as p53 does.

P53 gene encodes a 393 amino-acid protein which contains three important domains: an amino-terminal transactivation domain, a core domain which recognizes p53 DNA binding sites, and a carboxy-terminal tetramerization domain [5], [6]. About 75% of mutations are single amino acid substitutions in the core domain [7]. There are three (not mutually exclusive) kinds of outcomes when p53 mutation occurs [8], [9]. The first kind of mutation is to destroy the function of tumour suppressor for the affected allele of p53; if both alleles are mutated, the cells will completely loss the capacity of anticancer protection provided by p53. The second kind of mutation is to make the mutant p53 dominate the wild-type p53 by forming inactive mixed tetramers so as to deprive the ability of binding to DNA and transactivation. Therefore, even with one wild-type allele mutated, the cell may practically loss of the wild-type p53 function. The last kind of mutation is to make the mutant p53 gain or enhance its function for tumour progression [8], [9]. In other words, different kinds of p53 mutations may have completely different impacts to cancer patients. Accordingly, knowing mutant functional properties across a mutation sequence space is of specific interest that could advance medical practice. However, mutation spaces grow to be combinatorially large and hence making it time-consuming and labour-intensive for experimental studies. The resources for such experimental studies may also be quite limited. In view of this, it is important and urgent to develop computational approaches for studying the effects of different kinds of mutation or mutation-combinations, as well as the relevant features that dominate these effects.

The present study was devoted to develop a new computational method for predicting the active status of one-, two-, three- and four site p53 mutants. Our method used eight types of features: (1) gain/loss of amino acids during evolution [10] and conservation of amino acid at protein-protein interface [11]; (2) physicochemical and biochemical properties of amino acid, i.e., the “amino acid factors”; (3) conservation; (4) structural disorder; (5) distance between mutations; (6) the physicochemical differences between the original amino acid and the new amino acid at the mutation site; (7) 2D structure surface of the mutant protein; (8) 3D structure changes of the p53 protein caused by the mutation. The optimal features were selected based on the Maximum Relevance & Minimum Redundancy (mRMR) and Incremental Feature Selection (IFS). The Mathew's correlation coefficients (MCC) obtained by using Nearest Neighbor Algorithm (NNA) and jackknife cross validation for one-, two-, three- and four- site p53 mutants were 0.678, 0.314, 0.705 and 0.907, respectively. It has been revealed through further optimal feature set analysis that the 2D structure features composed the largest part of the optimal feature set and played the most important roles in all these four types of p53 mutant active status prediction. It has also been demonstrated via analysing the optimal feature sets, especially those at the top level, that the 3D structure features, PSSM conservation features and amino acid factor features played important roles in p53 mutant active status prediction.

According to a recent comprehensive review [12], to establish a really useful statistical predictor for a protein or peptide system, we need to consider the following procedures: (i) construct or select a valid benchmark dataset to train and test the predictor; (ii) formulate the protein or peptide samples with an effective mathematical expression that can truly reflect their intrinsic correlation with the attribute to be predicted; (iii) introduce or develop a powerful algorithm (or engine) to operate the prediction; (iv) properly perform cross-validation tests to objectively evaluate the anticipated accuracy of the predictor. Below, let us describe how to deal with these steps.

## Materials and Methods

### Dataset

We downloaded the mutant p53 transcriptional activity data set from UCI Machine Learning Repository http://archive.ics.uci.edu/ml/datasets/p53Mutants [13], [14], [15]. After filtering the mutations that could not be encoded, there were 62 one-site mutations (7 active ones, 55 inactive ones), 16372 two-site mutations (57 active ones, 16315 inactive ones), 111 three-site mutations (63 active ones, 48 inactive ones) and 31 four-site mutations (7 active ones, 24 inactive ones). We used the following eight types of features to encode the mutation site and its upstream/downstream four amino acids.

As mentioned above, to develop a powerful predictor for a protein or peptide system, one of the keys is to formulate the protein or peptide samples with an effective mathematical expression or vector that can truly reflect their intrinsic correlation with the target to be predicted. To realize this, let us utilize the general form of pseudo amino acid composition (PseAAC) [16] that can be formulated as follows [12]

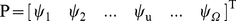
(1)where 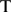 is a transpose operator, while the subscript 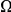 reflects the dimension of the vector and its value as well as the components 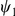, 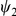, … will be defined by a series of feature extractions as elaborated below.

### Type 1 features: gain/loss of amino acids during evolution and conservation of amino acid at protein-protein interface

Let us consider the following two kinds of features: one representing the gain/loss of amino acids during evolution [10] (we called “SNP” feature for short), and the other representing conservation of amino acid at protein-protein interface [11] (hereafter it will be abbreviated as “pro-pro” feature) for each amino acid in each 9 amino-acid peptide. The SNP feature of gain/loss of amino acids during evolution was calculated based on the normalized differences between the number of substitutions creating and removing the amino acid [10]. The pro-pro feature of conservation of amino acid at protein-protein interface was calculated based on the number of conserved residues of this kind of amino acid at the contact interface, the total number of residues of this kind of amino acid in the protein, and the number of total amino acids in the protein [11]. It represents the conservation propensities on the binding sites [11].

### Type 2 features: physicochemical and biochemical features of amino acid

The diversity and specificity of protein structures and functions are largely attributed to the different compositions of different amino acids, which have different physicochemical properties. Atchley et al. [17] have performed multivariate statistical analyses on AAIndex [18] that is a database of various amino acid physicochemical and biochemical properties. These authors have summarized and transformed AAIndex to five highly compact numeric patterns to reflect the polarity (Factor 1), secondary structure (Factor 2), molecular volume (Factor 3), codon diversity (Factor 4), and electrostatic charge (Factor 5). In the current study, these five numerical pattern scores (abbreviated as “amino acid factors” or “AAFactor”) are used to represent the respective properties of each amino acid. Thus, there are 5 AAFactor features for each of the amino acids in a 9 amino-acid peptide.

### Type 3 features: conservation of residues

The position specific iterative BLAST (PSI BLAST) [19] was adopted to quantify the conservation probabilities of an amino acid against the 20 different types of native amino acids by using a 20D vector. All such 20D vectors for all residues in a given protein sequence formed the position specific scoring matrix (PSSM). Residues, which are more important for biological function, are more conserved through cycles of PSI BLAST. There are 20 PSSM features for each of the amino acids in a 9 amino-acid peptide.

### Type 4 features: structural disorder

The VSL2 [20] was used to score the structural disorder of each amino acid in the protein sequence. Protein disordered region is a segment that lacks 3D structures under physiological conditions and plays important roles in signalling control and regulation. There is one disorder feature for each of the amino acids in a 9 amino-acid peptide.

### Type 5 features: distance between mutation sites

In human mammary carcinoma, the mutation sites of p53 tend to occur within one single exon away or a short distance from another [21], implying that the distance of mutations may be of importance for affecting the function of p53. Here, we used 1, 2, 3 distance features for two-, three- and four-site p53 mutants respectively. The distance features represent the distance between adjacency mutations. For example, in three-site mutants, there were 2 distance features, which were the distance from the first mutant site to the second one, and the distance from the second to the third. Other distance features were defined in a similar way.

### Type 6 features: the physicochemical differences between the original amino acid and the new amino acid at the mutation site

The GRANTHAM score [22] was adopted to measure the physicochemical differences between two amino acids. According to such a score, if two amino acids have similar physicochemical features, the mutation from one to another will not cause the change of protein functions. There is one GRANTHAM feature for each mutant site.

### Type 7 features: 2D structure features

The structure features for each mutant were calculated based on the homology models [14], [15]. The structures of mutant proteins were simulated based on the structure of wild type p53 with mutant amino acids substituted. Then structure features were extracted from the energy minimized mutant model [13]. The attributes 1–4826 of structure features (V1–V4826) were calculated based on the 2D surface map of the mutant protein [13], [14], [15] where it is available for molecular interactions or drug binding.

### Type 8 features: 3D structure features

Attributes 4827–5408 (V4827–V5408) of structure features were calculated based on the 3D distance difference map between mutant and wild-type p53 [13], [14], [15]. Mutation of amino acid in p53 may change the 3D structure of protein. The 3D distance map of p53 protein is an N×N matrix showing the distance between N residue alpha carbons [15]. It reflects the structural changes caused by the mutation of amino acids. After subtracting the distance map of wild-type p53, a difference map was obtained. The 3D distance difference map features represent the magnitudes of the distance changes in 3D structure. Both the 2D structure features and 3D structure features were downloaded from UCI Machine Learning Repository http://archive.ics.uci.edu/ml/datasets/p53Mutants [13], [14], [15].

### Feature space for one-site, two-site, three-site and four-site mutants

Shown in Table 1 is a breakdown of the number of each kind of features for one-site, two-site, three-site and four-site mutants, respectively. Accordingly, we totally have 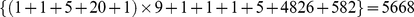 features for a 9 amino acid peptide with one-site mutant. Similarly, we have 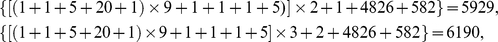
and 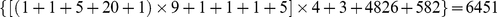 features for a 9 amino acid peptide with one-site, two-site, and four-site mutants, respectively. Accordingly, the dimension 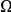 for a 9 amino acid peptide mutant as formulated by equation (1) can now be expressed by
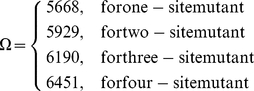
(2)


**Table 1:** Number of features for one-site, two-site, three-site and four-site mutants.

Features	One-site mutant	Two-site mutant	Three-site mutant	Four-site mutant
SNP features^a^	1×9+1 = 10	(1×9+1)×2 = 20	(1×9+1)×3 = 30	(1×9+1)×4 = 40
Pro-pro features^b^	1×9+1 = 10	(1×9+1)×2 = 20	(1×9+1)×3 = 30	(1×9+1)×4 = 40
Amino acid factor	5×9+5 = 50	(5×9+5)×2 = 100	(5×9+5)×3 = 150	(5×9+5)×4 = 200
PSSM features	20×9 = 180	20×9×2 = 360	20×9×3 = 540	20×9×4 = 720
Disorder feature	1×9 = 9	1×9×2 = 18	1×9×3 = 27	1×9×4 = 36
GRANTHAM	1	2	3	4
Distance features	0	1	2	3
2D structure features	4826	4826	4826	4826
3D structure features	582	582	582	582
Total	5668	5929	6190	6451

a Gain/loss of amino acids during evolution.

b Conservation of amino acid at protein-protein interface.

Thus, substituting the dimension value for 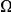 as well as the value for each of relevant features as described above into equation (1), we immediately obtain the 5668D, 5929D, 6190D, or 6451D vector for the one-site, two-site, three-site, or four-site mutant of 9 amino acid peptide, respectively. The vectors thus obtained will be used to represent the statistical samples concerned for the current study.

### mRMR method

To rank the features with their importance, we used the maximum relevance minimum redundancy (mRMR) method developed by by Peng et al. [23]. The mRMR program can be downloaded from http://penglab.janelia.org/proj/mRMR/. It recursively selects the feature that has the maximum relevance to the target variable and minimum redundancy to the already selected features. Features that have a smaller index mean that they are selected earlier and are more important. We used the mutual information (MI) to quantify the relation between two vectors, which was defined as follows
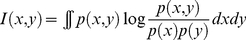
(3)


In equation (3), 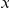 and 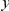
denote vectors; 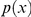 and 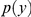 denote the marginal probabilistic densities; and 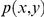 denotes joint probabilistic density.

To quantify both relevance and redundancy, we defined 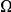 as the whole feature set, 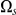 as the already-selected feature set containing m features and 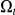 as the to-be-selected feature set containing n features. The relevance 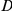 between feature 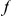 in 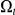 and the target 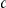 can be calculated by:
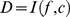
(4)


The redundancy 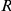 between the feature 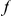 in 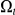 and all the features in 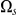 can be calculated by:
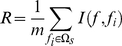
(5)


The mRMR function, which combined equation (4) and equation (5) and can be used to obtain the feature 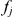 in 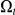 with maximum relevance and minimum redundancy, was defined as following:
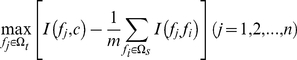
(6)


Given a feature set with 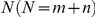 features, the feature evaluation will be performed *N* rounds. After these evaluations, mRMR method will generate a feature set 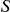:
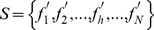
(7)


In this feature set 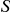, each feature has an index h, indicating which round the feature is selected. A better feature will be selected earlier and have a smaller index *h*.

### Nearest Neighbor Algorithm

We used nearest neighbor algorithm (NNA) [12] to build the prediction model of p53 activity. NNA calculates similarities between the test sample and all the training samples. In the current study, the distance between vector 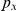 and 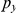 is defined as following [24], [25], [26], [27], [28], [29], [30], [31]:
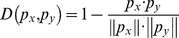
(8)


In equation (8), 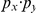 denotes the inner product of 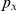 and 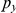. 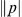 denotes the module of vector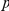. The smaller 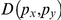 is, the more similar 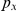 to 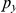 is.

In NNA, given a vector 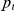
and training set 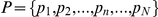, 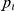 will be designated to the same class of its nearest neighbor 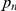 in 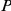, i.e. the vector having the smallest 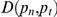:
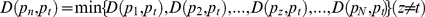
(9)


### Jackknife Cross-Validation Method

In statistical prediction, the following three cross-validation methods are often used to examine a predictor for its effectiveness in practical application: independent dataset test, subsampling test, and jackknife test [32]. However, as elucidated in [33] and demonstrated by Eqs.28–32 of [12], among the three cross-validation methods, the jackknife test is deemed the least arbitrary that can always yield a unique result for a given benchmark dataset, and hence has been increasingly used and widely recognized by investigators to examine the accuracy of various predictors (see, e.g., [34], [35], [36], [37], [38], [39], [40], [41]). Accordingly, in this study we also used the jackknife test to evaluate the performance of our classifier. In the jackknife cross-validation, each of the statistical samples in the benchmark dataset is in turn singled out as a tested sample and the predictor is trained by the remaining samples. During the jackknifing process, both the training dataset and testing dataset are actually open, and a statistical sample will in turn move from one to the other. The jackknife cross-validation can exclude the memory effects during entire testing process and also the result thus obtained is always unique for a given benchmark dataset [25].

Since the positive and negative samples are highly imbalanced in the data set, the Matthews's correlation coefficient (MCC) [42] was used to evaluate the prediction performance and its definition is given by
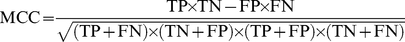
(10) where TP, TN, FP and FN were the number of true active mutants, true inactive mutants, false active mutants and false inactive mutants, respectively [43].

### Incremental Feature Selection

With features ranked by mRMR method, incremental feature selection (IFS) was applied to determine the optimal number of features [26], [27], [28], [29], [30], [44]. An incremental feature selection is conducted for each of the independent predictor with the ranked features. Features in a set are added one by one from higher to lower rank. If one feature is added, a new feature set is obtained, then we get N feature sets, and the i-th feature set is:
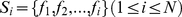
(11) where *N* is the number of features. With each of the *N* feature sets, an NNA predictor was constructed and tested using Jackknife cross-validation test. With MCC of jackknife cross-validation calculated, we obtain an IFS table with the number of features and the performance of them. 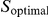 is the optimal feature set that achieves the highest MCC.

## Results

### mRMR result

Using the mRMR program, we obtained the ranked mRMR list of 5668, 5929, 6190 and 6451 features for one-, two-, three- and four-site p53 mutants respectively (cf. Eq.2). Within the lists, the smaller index of a feature indicates it has a more important role in discriminating positive samples from negative ones. The mRMR lists were used in IFS procedure for further feature selection and analysis.

### IFS result

**Figure 1:**
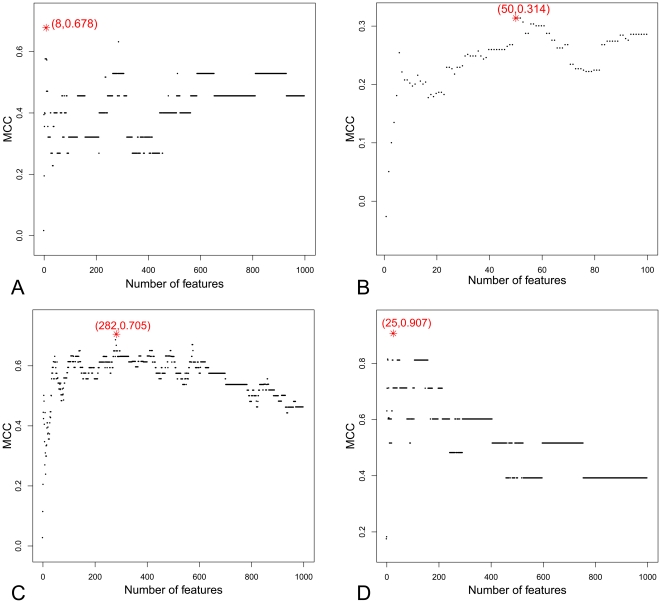
The IFS curves for one-site, two-site, three-site and four-site p53 mutants. In the IFS curve, the x-axis is the number of features used for classification, and the y-axis is the Mathew's correlation coefficients (MCC) generated by the jackknife test. (A) The IFS curve for one-site p53 mutants. The peak of MCC is 0.678 with 8 features. The top 8 features derived by the mRMR approach form the optimal feature set for one-site p53 mutants. (B) The IFS curve for two-site p53 mutants. The peak of MCC is 0.314 with 50 features. The top 50 features derived by the mRMR approach form the optimal feature set for two-site p53 mutants. (C) The IFS curve for three-site p53 mutants. The peak of MCC is 0.705 with 282 features. The top 282 features derived from the mRMR approach form the optimal feature set for three-site p53 mutants. (D) The IFS curve for four-site p53 mutants. The peak of MCC is 0.907 with 25 features. The top 25 features derived from the mRMR approach form the optimal feature set for four-site p53 mutants.

Based on the outputs of mRMR, we built individual predictors by adding features recursively from the top of the mRMR output to the bottom to predict the active status of p53 mutants. We tested each of the individual predictors and obtained the IFS results. The IFS results for one-, two-, three- and four-site p53 mutants are provided as Table S1, Table S2, Table S3 and Table S4, respectively. The IFS curves for one-site, two-site, three-site and four-site p53 mutants were shown in Figure 1.

### Optimal feature set analysis for one-site p53 mutants

Figure 1 A shows IFS curve plotted based on Table S1. The maximum MCC is 0.678 by using 8 features as shown in Table 2.

**Table 2:** Optimal feature set for one-site p53 mutants.

Order	Name	Score
1	AA3_PSSM-8-G	0.144
2	AA8_PSSM-19-Y	0.105
3	V241	0.067
4	AA6_AAFactor-3	0.052
5	V78	0.05
6	AA5_AAFactor-1	0.04
7	AA2_PSSM-18-W	0.039
8	AA4_disorder	0.04

Within the optimal feature set for one-site p53 mutants active status prediction, there are two 2D structure features (V241 and V78), three PSSM features (i.e., AA3_PSSM-8-G: the conservation status against G at residue 3; AA8_PSSM-19-Y: the conservation status against Y at residue 8; and AA2_PSSM-18-W: the conservation status against W at residue 2), two AAFactor features (AA6_AAFactor-3: the molecular volume amino acid factor feature at residue 6; AA5_AAFactor-1: the polarity amino acid factor feature at residue 6) and one disorder feature at residue 4.

### Optimal feature set analysis for two-site p53 mutants

Shown in Figure 1 B is the IFS curve plotted based on Table S2. The maximum MCC is 0.314 by using 50 features. The 50 optimal features for the two-site p53 mutants are given in Table S5.

Within the top 50 features, there are 49 2D structure features and 1 PSSM feature (AP2.AA8_PSSM-3-N). Listed in Table 3 are the top 10 features for two-site p53 mutants. The conservation status against *N* at residue 8 is the top feature within the selected optimal feature set, indicating that site 8 and the specific mutation status against *N* plays the most important role in determining the active status of two-site p53 mutants. The inclusion of 49 2D structure features within the optimal 50 features indicates its important roles. As mentioned in introduction, the majority of p53 mutations occurred in the core DNA-binding domain [7]. Within the DNA-binding domain of p53 protein, the secondary structures (the two alpha-helices and the eleven beta-strands) were susceptible to amino acid substitution [45]. Thus, the changes of secondary structures would alter the DNA contact and Zn binding so as to cause functional changes [45].

**Table 3:** Top 10 features for two-site p53 mutants.

Order	Name	Score
1	AP2.AA8_PSSM-3-N	0.004
2	V1152	0.002
3	V55	0.002
4	V1854	0.001
5	V4001	0
6	V2846	0
7	V4168	0
8	V1059	0
9	V2633	0
10	V3105	0

### Optimal feature set analysis for three-site p53 mutants

Shown in Figure 1 C is the IFS curve plotted based on Table S3. The maximum MCC is 0.705 using 282 features although it can reach 0.632 already with the top 48 features. The 282 optimal features for three-site p53 mutants are provided in Table S6. There are totally 249 structure features (including 214 2D features, 35 3D structure features), 22 PSSM features, 7 AAFactor features, 1 SNP feature, 2 disorder features, and 1 pro-pro feature.

The top 10 features for active status prediction of three-site p53 mutants are shown in Table 4. There are 7 2D structure features (including the Top four features), indicating that they have the most important impact on the three-site p53 mutants active status prediction. The fifth feature is the polarity amino acid factor feature at residue 2 and mutation site 1 (AP1.AA2_AAFactor-1). This indicates that residue 2 at mutation site 1, especially its polarity property, would play an important role for the active status prediction of three-site p53 mutants. The 3D structure features (index 6 and 8) may also play some roles for this type of prediction.

**Table 4:** Top 10 features for three-site p53 mutants.

Order	Name	Score
1	V2261	0.159
2	V3291	0.074
3	V4391	0.069
4	V3106	0.067
5	AP1.AA2_AAFactor-1	0.056
6	V5068	0.061
7	V4075	0.049
8	V5278	0.046
9	V3568	0.05
10	V3978	0.052

There are 18, 9, and 6 optimal features at mutation site 1, 2, and 3, respectively. For more detailed information about these optimal features, please refer to Table S7, S8 and S9, respectively.

### Optimal feature set analysis for four-site p53 mutants

As we can see from Figure 1 D, the MCC reached the maximum value (0.907) when using 25 features shown in Table 5.

**Table 5:** Optimal feature set for four-site p53 mutants.

Order	Name	Score
1	V431	0.461
2	V4965	0.109
3	V1675	0.147
4	V414	0.132
5	V3945	0.097
6	V1116	0.102
7	V2789	0.1
8	V407	0.097
9	AP1.AA9_PSSM-7-E	0.09
10	V432	0.084
11	V3562	0.08
12	V4524	0.079
13	V2253	0.077
14	AP1.AA2_PSSM-11-L	0.077
15	V1099	0.071
16	V2718	0.067
17	V438	0.07
18	V4946	0.07
19	V2817	0.069
20	V1159	0.072
21	V3477	0.072
22	V2357	0.07
23	V415	0.07
24	AP1.AA2_AAFactor-4	0.072
25	AP2.AA1_PSSM-14-F	0.072

In the optimal feature set there are three AP1 features (AP1.AA9_PSSM-7-E, AP1.AA2_PSSM-11-L, and AP1.AA2_AAFactor-4) as well as one AP2 feature (AP2.AA1_PSSM-14-F), indicating that these specific features at relevant residues may play more roles than other features and residues.

The optimal feature set also contains 19 2D structure features and 2 3D structure features (including the top 8 features), which is fully consistent with the majority of this optimal feature set (21/25), indicating that these two types of features would play important roles in the four-site p53 mutant active status prediction.

### Comparison of the optimal feature sets of the four types of p53 mutants

By comparison of the optimal feature sets for one-, two-, three- and four-site p53 mutants, we can now see that the 2D structure features composed the largest part of the optimal feature set and hence might play the most important roles in all these four types of p53 mutant active status prediction. It has also been demonstrated through the optimal feature sets (especially those at the top level) that the 3D structure features, PSSM conservation features and AAFactor features did play important roles in p53 mutant active status prediction. The selected optimal feature sets, especially those at the top level, may provide important clues or insights for further experimental studies in this area.

## Discussion

### The relationship between structure change and function change in p53 mutants

It was found through this study that most of the selected features were those directly related to structure. The relationship between structure and function of p53 were suspected for quite a long time. Most cancer-associated p53 amino acid mutations are located at the highly conserved central DNA binding domain, suggesting a correlation between the evolutionary conservation and the structural or functional importance of amino acid residues [45], [46]. It has been reported [45], [47] that those residues, which are in contacting with DNA or located at the opposite side of DNA, would form the core of the folded protein, and hence are most likely to be conserved and mutated. Most hotspots with high evolutionary conservation are either near to the DNA-protein interface, or at the amino acids in contacting with DNA [45], [47]. Mutation of cysteines 176, 238 and 242 to serine within the zinc region will completely block the transcriptional activation of p53 [48]. It is evidenced that mutation of arginine 156, arginine 158, serine 215 and glutamate 258 in p53 protein will destabilize the protein structure [45], [48] owing to the repulsion interactions between the side chains of these amino acids.

### The imbalance of features from different sites in multiple-site p53 mutants

It has been observed through this study that the selected features of multiple-site p53 mutants are usually located on only part of the mutation sites. This is probably due to the reason that the unselected mutation sites might contribute nothing to the p53 functional abnormality because the selected mutation sites, which serve as “hitch-hikers” [49], have already done the job.

## Supporting Information

Table S1 The IFS results for one-site p53 mutants.
(XLS)

Table S2 The IFS results for two-site p53 mutants.
(XLS)

Table S3 The IFS results for three-site p53 mutants.
(XLS)

Table S4 The IFS results for four-site p53 mutants.
(XLS)

Table S5 The 50 optimal features for two-site p53 mutants.
(XLS)

Table S6 The 282 optimal features for three-site p53 mutants.
(XLS)

Table S7 The 18 optimal features at mutation site 1 for three-site p53 mutants.
(XLS)

Table S8 The 9 optimal features at mutation site 2 for three-site p53 mutants.
(XLS)

Table S9 The 6 optimal features at mutation site 3 for three-site p53 mutants.
(XLS)
